# Performance of a High-Sensitivity Rapid Diagnostic Test for *Plasmodium falciparum* Malaria in Asymptomatic Individuals from Uganda and Myanmar and Naive Human Challenge Infections

**DOI:** 10.4269/ajtmh.17-0245

**Published:** 2017-08-07

**Authors:** Smita Das, Ihn Kyung Jang, Becky Barney, Roger Peck, John C. Rek, Emmanuel Arinaitwe, Harriet Adrama, Maxwell Murphy, Mallika Imwong, Clare L. Ling, Stephane Proux, Warat Haohankhunnatham, Melissa Rist, Annette M. Seilie, Amelia Hanron, Glenda Daza, Ming Chang, Tomoka Nakamura, Michael Kalnoky, Paul Labarre, Sean C. Murphy, James S. McCarthy, Francois Nosten, Bryan Greenhouse, Sophie Allauzen, Gonzalo J. Domingo

**Affiliations:** 1 Diagnostics Program, PATH, Seattle, Washington;; 2 Infectious Disease Research Collaboration, Kampala, Uganda;; 3 University of California San Francisco, San Francisco, California;; 4 Department of Molecular Tropical Medicine and Genetics, Faculty of Tropical Medicine, Mahidol University, Bangkok, Thailand;; 5 Shoklo Malaria Research Unit, Mahidol-Oxford Tropical Medicine Research Unit, Faculty of Tropical Medicine, Mahidol University, Mae Sot, Thailand;; 6 QIMR Berghofer Medical Research Institute, Brisbane, Australia;; 7 Department of Laboratory Medicine, University of Washington, Seattle, Washington;; 8 Department of Microbiology, University of Washington, Seattle, Washington;; 9 Center for Emerging and Re-emerging Infectious Diseases, University of Washington, Seattle, Washington;; 10 Centre for Tropical Medicine and Global Health, Nuffield Department of Medicine, University of Oxford, Oxford, United Kingdom;; 11 Bill & Melinda Gates Foundation, Seattle, Washington

## Abstract

Sensitive field-deployable diagnostic tests can assist malaria programs in achieving elimination. The performance of a new Alere™ Malaria Ag P.f Ultra Sensitive rapid diagnostic test (uRDT) was compared with the currently available SD Bioline Malaria Ag P.f RDT in blood specimens from asymptomatic individuals in Nagongera, Uganda, and in a Karen Village, Myanmar, representative of high- and low-transmission areas, respectively, as well as in pretreatment specimens from study participants from four *Plasmodium falciparum*-induced blood-stage malaria (IBSM) studies. A quantitative reverse transcription PCR (qRT-PCR) and a highly sensitive enzyme-linked immunosorbent assay (ELISA) test for histidine-rich protein II (HRP2) were used as reference assays. The uRDT showed a greater than 10-fold lower limit of detection for HRP2 compared with the RDT. The sensitivity of the uRDT was 84% and 44% against qRT-PCR in Uganda and Myanmar, respectively, and that of the RDT was 62% and 0% for the same two sites. The specificities of the uRDT were 92% and 99.8% against qRT-PCR for Uganda and Myanmar, respectively, and 99% and 99.8% against the HRP2 reference ELISA. The RDT had specificities of 95% and 100% against qRT-PCR for Uganda and Myanmar, respectively, and 96% and 100% against the HRP2 reference ELISA. The uRDT detected new infections in IBSM study participants 1.5 days sooner than the RDT. The uRDT has the same workflow as currently available RDTs, but improved performance characteristics to identify asymptomatic malaria infections. The uRDT may be a useful tool for malaria elimination strategies.

## INTRODUCTION

Nearly half of the world’s population remains at risk for malaria, despite substantial gains in morbidity and mortality from 2001 to 2015.^1^ The majority of the 212 million cases reported in 2015 were attributed to *Plasmodium falciparum*, with the greatest incidence in sub-Saharan Africa (90%) followed by the southeast Asia region (7%).^1^ Since 2000, the scale up of interventions such as vector control, rapid diagnostic tests (RDTs), and artemisinin-based combination therapies (ACTs) have resulted in a significant decrease in malaria cases globally,^1^ such that in 2015, 17 countries eliminated malaria.^1^ Country or regional elimination is defined as zero indigenous malaria cases caused by a particular *Plasmodium* species for 3 years or more.^1,2^ As countries aim to reach this goal, the inability of current diagnostic tools to detect asymptomatic low-density infections has become apparent and the lack of such tools has consequently hindered such efforts.^3–5^ The ability of individuals to carry low-density malaria infections and have minimal clinical symptoms is believed to be the consequence of exposure-related clinical immunity, resulting in the suppression of parasitemia, and has been observed in both high- and low-transmission settings.^3,5,6^ These infections can have a long duration. Studies relating gametocyte density to parasite density, mosquito feed studies that have shown human to mosquito transmission in low-density infections, as well as modeling suggest that these low chronic infections have a potential to contribute significantly to ongoing transmission in a community.^4,7,8^ As a result, asymptomatic individuals with chronic infection remain reservoirs that contribute to onward transmission and represent a serious concern for elimination efforts.^9–12^

Some elimination programs use active case detection (ACD) strategies such as mass or focal test-and-treat following an index case identified at a health clinic. ACD is performed outside of health clinics and is largely dependent on rapid diagnosis by the two most commonly used field tools: microscopy and/or RDTs.^10–13^ Although these two tests have been largely successful in control efforts for case management, they have failed to identify malaria infections among significant proportions individuals infected with malaria but presenting with no clinical symptoms. For example, in Zambia, where the *P. falciparum* prevalence was less than 10% from 2009 to 2012, the sensitivity of RDT compared with nested polymerase chain reaction (PCR) in a reactive case detection strategy was only 17% in individuals with asymptomatic infections.^14^ Additionally, in western Kenya, when children and adolescents with no clinical symptoms in highland (low-transmission) and lowland (high-transmission) areas were tested for *P. falciparum* malaria by microscopy and quantitative PCR (qPCR), the average microscopy-based prevalence was 13.3%, whereas the qPCR-based prevalence was 20.9%, an almost two-fold difference.^5^ Similar variability in malaria prevalence by microscopy and/or RDT versus qPCR has been also reported in South America and southeast Asia, where the rates of asymptomatic infections were four to five times that of symptomatic infections.^3,9,15–20^ These data suggest that a large proportion of individuals who have no clinical symptoms of malaria, but are infected, have low-density infections. A challenge in interpretation of these ratios is also the variability in the sensitivity of the various PCR methodologies applied. To support ACD strategies that reduce the hidden burden of malaria and make elimination feasible, a more sensitive, field-deployable *P. falciparum* malaria infection detection test is required.^4,12,14^

The quantification of *P. falciparum* biomarkers in asymptomatic subpatent infections in the field is important for establishing the required limit of detection (LOD) for a highly sensitive diagnostic test. One of the most commonly used tools, microscopy, identifies both gametocytes and asexual parasites and can achieve an LOD of 4–20 parasites/µL (p/µL) in laboratories with expert microscopists and approximately 200 p/µL in field conditions with inexperienced microscopists.^4,10,13,21^ In addition, microscopy lacks reproducibility and has variable sensitivity and a high false-positive rate.^22^ The recent introduction of RDTs has changed the malaria diagnostics landscape, allowing rapid diagnosis and treatment in both clinical and nonclinical settings. However, it has a detection threshold similar to that of microscopy and therefore is largely useful only in diagnosing symptomatic malaria.^4,23^ Molecular laboratory-based methods such as qPCR and quantitative reverse-transcription PCR (qRT-PCR) enable detection of low levels of parasite nucleic acids (often with LODs < 1 p/µL), and have helped distinguish and quantify low-density infections missed by microscopy and RDTs.^4,5^ A recently developed histidine-rich protein II (HRP2) enzyme-linked immunosorbent assay (ELISA) with an established laboratory LOD of 5 picograms/mL (pg/mL) permits the evaluation of new more sensitive HRP2-based RDTs based on HRP2 concentration. Accordingly, measuring *P. falciparum* biomarkers using current high-performance diagnostics will generate fundamental data about biomarker concentrations in asymptomatic active infections and subsequently inform the diagnostic LOD required to detect the reservoir population.

Although laboratory assays are more sensitive than microscopy and RDTs, they are also more expensive, require highly trained personnel, take longer to produce results, and are more difficult to deploy for large-scale field use. An Alere^™^ Malaria Ag P.f Ultra Sensitive RDT (uRDT) was developed for field use and evaluated for detection of low-density infections. Compared with the current commercial HRP2-based SD BIOLINE Malaria Ag P.f. RDT (RDT), the uRDT uses the same immunochromatographic cassette platform, has the same whole blood volume requirements (5 µL), and takes only 5 minutes longer to result, making this device a promising and highly sensitive field tool. Currently, the best *P. falciparum* malaria RDTs have a LOD in the range of 600–1,000 pg/mL HRP2.^24,25^ The uRDT has an order of magnitude improvement in LOD for HRP2 (Peck et al., 2017, manuscript in preparation), such that overall, the uRDT may fulfill many of the necessary characteristics for field use, but has not yet been assessed against asymptomatic infections in malaria-endemic areas. Furthermore, the proportion of infections detected by the uRDT compared with RDT during the course of infection has not been explored. Although *P. falciparum*-induced blood-stage malaria (IBSM) human challenge studies are traditionally performed to measure vaccine or drug efficacy and pharmacodynamics, they can also inform the impact of LOD on early detection of a new infection by observing parasitemia and HRP2 concentrations by highly sensitive qRT-PCR and ELISA methods, respectively.^26^ For example, in a clinical vaccine trial, PCR with a test sensitivity of approximately 20 p/mL detected malaria infection 5 days earlier than thick smears read by expert microscopists in both naive and previously exposed volunteers.^27^

In this study, the new uRDT was evaluated for performance using whole blood specimens collected from asymptomatic individuals in Nagongera, Tororo District, Uganda, and a Karen village (TOT), Myanmar, representing high- and low-transmission settings, as well as from naive individuals enrolled in *P. falciparum* IBSM challenge studies.

## MATERIALS AND METHODS

### Human subjects research.

All specimens were collected from consenting study participants or with the assent of children and consenting legal care givers within the context of studies approved by the relevant institutional review boards (IRBs). Specimens from Uganda were collected under a study approved by the University of California San Francisco (UCSF) (IRB No.11-05995), Makerere University (IRB No. 2011-0167), and London School of Hygiene and Tropical Medicine (LSHTM) (IRB No. 5943); those from Myanmar were collected under a study approved by OxTREC (reference no. 1017-13 and 1015-13), by the Tak Community Advisory Board, and by the relevant village committees. The IBSM challenge studies were approved by the Queensland Institute for Medical Research (QIMR) (IRB No. 2080, 2092, 2098, 2142). Specimens were received at PATH as delinked and anonymized for analysis as per study consent forms.

### IBSM *P. falciparum* challenge studies.

IBSM challenge studies were performed at QIMR, Queensland, Australia. Whole blood specimens were collected from enrolled human volunteers across four IBSM *P. falciparum* studies performed in 2015 (trial registrations: NCT02389348, [*N* = 3], NCT02431637 [*N* = 2], NCT02431650 [*N* = 4], NCT02573857 [*N* = 7]).

All participants were deemed healthy and had not been previously exposed to malaria. Data are presented on specimens collected Days 0–7. Participants were inoculated intravenously with approximately 1,800–2,800 3D7 *P. falciparum* parasitized red blood cells on Day 0. On Days 0 and 4, blood was collected once from each participant, and then collected twice daily at 12-hour intervals from Day 5 to Day 6.5. On Day 7, participants were admitted to the study unit, treated with an antimalarial, and observed for at least 72 hours. If participants were clinically well, then outpatient monitoring occurred to ensure safety and clearance of parasites until discharge from the study.

### Clinical research sites.

Specimens for performance evaluations of the uRDT were collected at two clinical research sites: Nagongera, Uganda, and a Karen village (TOT), Myanmar. In addition, *P. falciparum* IBSM studies were performed in Queensland, Australia. For the Uganda and Myanmar uRDT studies, the inclusion criteria for asymptomatic individuals were no fever history in the previous 7 days, axillary temperature less than 37.5°C, the absence of other clinical signs of malaria, and no malaria treatment within the previous 60 days.

In Nagongera, Tororo District, Uganda, 100 random households were selected and visited to recruit children 6 months to 11 years old, as well as their primary caregivers, into a surveillance cohort as previously described.^28^ All participants provided informed consent. As part of the ongoing cohort study, study participants were required to visit a health clinic every 3 months for routine visits, at which time blood was collected by venipuncture and microscopy was performed. For this study, samples were taken during 2 consecutive routine visits from May to October 2015.

In TOT, Myanmar, study teams visited households and recruited both children and adults as part of an ongoing study to assess mass drug administration (MDA) for malaria elimination. All participants provided informed consent. Pregnant women and children less than 6 months of age were excluded.

### Blood collection and handling.

At all field sites and the IBSM site, 1–2 mL of whole blood was collected from each study participant by venipuncture into a labeled ethylenediaminetetraacetic acid (EDTA) vacutainer tube. The vacutainer was inverted gently several times to ensure adequate anticoagulation. For the field studies, whole blood specimens were then transported to a local laboratory in insulated containers containing conditioned ice packs or ambient temperature. Upon arrival at the laboratory, blood specimens were processed within 6–24 hours as per external study partners’ instructions for molecular and antigen detection testing. After processing, specimens were immediately stored at −80°C. If specimens could not be processed in the same day, then specimens were stored at 4°C for a maximum of 12 hours.

### *Plasmodium falciparum* parasite density determined by qRT-PCR.

At each site’s laboratory, whole blood specimens were prepared for qRT-PCR by aliquoting and mixing 50 µL of EDTA-anticoagulated whole blood into each of two tubes containing 2 mL NucliSENS lysis buffer (bioMérieux Inc., Marcy-lÉtoile, France). Fifty microliters of specimen were also added to each of five spots on a standard dried blood spot (DBS) card (Whatman 903 protein saver card; GE/Whatman, Marlborough, MA), then dried, desiccated, and frozen in gas-impermeable bags. The lysis buffer tubes and DBS cards were then frozen onsite and stored at −80°C until shipping. The University of Washington performed RNA nucleic acid extraction as previously described.^29,30^ qRT-PCR was subsequently performed using a multiplex assay for *P. falciparum* and pan-*Plasmodium*-specific 18S rRNA amplicons using a SensiFAST^™^ Probe Lo-ROX One-Step Kit (Bioline, Taunton, MA).^31^ Absolute copy number quantification was performed against an in vitro-transcribed RNA standard curve in a malaria-negative whole blood eluate and copy numbers were converted to estimated p/mL as described^30^ parasite density estimates are based on the 18S rRNA content of synchronized ring-stage *P. falciparum* parasites and the 18S rRNA-to-parasite conversion factor may vary for non-*P. falciparum* species. qRT-PCR-positive specimens were classified as *P. falciparum*, *P. falciparum* or mixed, non-*P. falciparum*, and *Plasmodium* spp.

The Myanmar specimens were also examined with the high-volume qPCR at Mahidol University as described.^32,33^ For high-volume qPCR-positive blood samples, malaria parasite species were identified using nested PCR specific for *P. falciparum* (microsatellite marker Pk2), *P. vivax* (microsatellite marker 3.502), and *Plasmodium malariae* (18S rRNA) as described previously.^32,33^

### *P. falciparum* HRP2 quantification by Q-plex ELISA.

At PATH, *P. falciparum* HRP2 and human C-reactive protein (CRP) were quantified in collected whole blood specimens using a recently developed Q-plex array ELISA kit (Quansys Biosciences, Logan, UT). The measurement of HRP2 and CRP used a sandwich ELISA and competitive ELISA methods, respectively. For *P. falciparum* HRP2 and CRP, the analytical LODs are 5 pg/mL and 25 ng/mL, respectively. Only the *P. falciparum*-specific HRP2 data are presented herein.

The *P. falciparum* HRP2 Q-plex ELISA was performed according to the manufacturer’s kit instructions. The competitor for CRP and calibrator for *P. falciparum* HRP2 were prepared in an 8-point 3-fold standard dilutions with sample diluent containing heterophilic antibodies and rheumatoid factor blockers. Positive and negative controls and a blank were also included on each plate. The positive control was *P. falciparum* ITG (recombinant HRP2)-spiked negative North American whole blood, the negative was North American whole blood only, and the blank was standard diluent. Each specimen was tested in duplicate. To each plate well, 12.5 µL of whole blood and 37.5 µL of sample diluent were added, to make a total volume of 50 µL. The plate was then sealed, wrapped with foil, and placed on a shaker at 500 rpm for 1 hour at room temperature. After washing the plate three times with 1× wash buffer, 50 µL of detection mix containing biotinylated detector antibodies was added to each well and incubated on a shaker (500 rpm) for 20 minutes. The plate was washed three times followed by the addition of 50 µL of 1× streptavidin–horseradish peroxidase (HRP), incubated again for 20 minutes on the shaker (500 rpm), and then washed six times. Equal volumes of substrate A (hydrogen peroxide) and substrate B (signal enhancer) were mixed, of which 50 µL was then added to each well. The plate was then imaged using a Q-view imager (Quansys Biosciences, Logan, UT). Quality control specified for replicates a < 15% variation, and the blank and negative control wells had a capture image pixel intensity value ≤150. The analytical LOD of *P. falciparum* HRP2 in the Q-plex array, 5 pg/mL, was used as the positive–negative threshold for all specimens. The upper LOD was 14,600 pg/mL. The Five-Parameter Logistics model with weighting was used to interpolate the data based on the standard curve and determine *P. falciparum* HRP2 concentration in each specimen.

### *P. falciparum* detection by RDT and uRDT.

All whole blood specimens were tested in singlet using the Standard Diagnostics, Inc. (SD) BIOLINE Malaria Ag P.f. RDT (SD/Alere, Yongin-si, Republic of Korea, Cat. 05EK50) and in duplicate using Alere Malaria Ag P.f RDT ULTRA SENSITIVE (SD/Alere, Yongin-si, Republic of Korea) according to the manufacturer’s instructions. Briefly, the RDT and uRDT were stored and used at room temperature, and blood specimens were defrosted and placed on ice prior to use. For both RDTs, 5 µL of whole blood was transferred by calibrated pipette to the round specimen well, followed by the addition of four drops of diluent into the square well. The test results were read at 15 minutes for the RDT and at 20 minutes for the uRDT. The results were interpreted as invalid (no control line), positive (control line and test line present), or negative (control line present, no test line).

### Statistical analysis.

The histograms depicting the distributions of parasite density and HRP2 in asymptomatic infections and uRDT detection from Uganda, Myanmar, and *P. falciparum* IBSM challenge model were produced using Microsoft Excel 2013 (Microsoft, Redmond, WA). The sensitivities of the RDT and uRDT against qRT-PCR or Q-plex ELISA reference tests were calculated as true positives/(true positives + false negatives), and specificity calculated as true negatives/(true negatives + false positives). The positive and negative predictive values were calculated as true positives/(true positive + false positives) and true negatives/(true negatives + false negatives), respectively. For the IBSM studies, two-tailed *Z* tests with a significance level of 0.05 were performed to test the null hypothesis that the proportion of uRDT positives was equivalent to that of the RDT positives.

## RESULTS

### Specimen collection.

Across four *P. falciparum* IBSM studies, a total of 93 whole blood specimens from Day 4 to Day 7, comprising the time points prior to treatment (Day 7), were collected from 16 human volunteers. In Myanmar, 493 whole blood specimens comprising all individuals residing in the Karen village were collected from April 21 to May 7, 2015. In Uganda, a total of 607 specimens were collected.

### Parasite density and HRP2 concentration.

All parasite density estimates for this study were determined by qRT-PCR and described in p/µL. HRP2 concentration was determined using the Q-plex ELISA, which has a LOD of 5 pg/mL GST-W2 recombinant HRP2 (Jang et al., 2017, manuscript in preparation), and described in pg/mL.

#### Pretreatment specimens in P. falciparum IBSM challenge studies.

**Figure 1.**
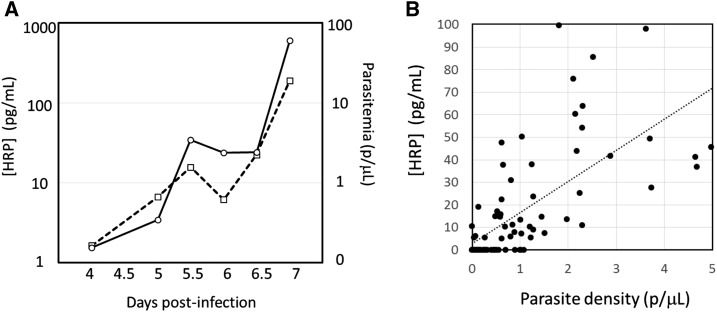
Relationship between parasite density and histidine-rich protein II (HRP2) concentration in IBSM studies. (**A**) HRP2 concentration and parasite density profiles over days after infection with parasitized red blood cells. Dashed line and empty squares represent parasite density; solid line and empty circles represent HRP2 concentration. The means of results from 16 subjects are shown. (**B**) Relationship between parasite density and HRP2 concentration in the 0–5 parasites per µL range pre-treatment infection.

**Figure 2.**
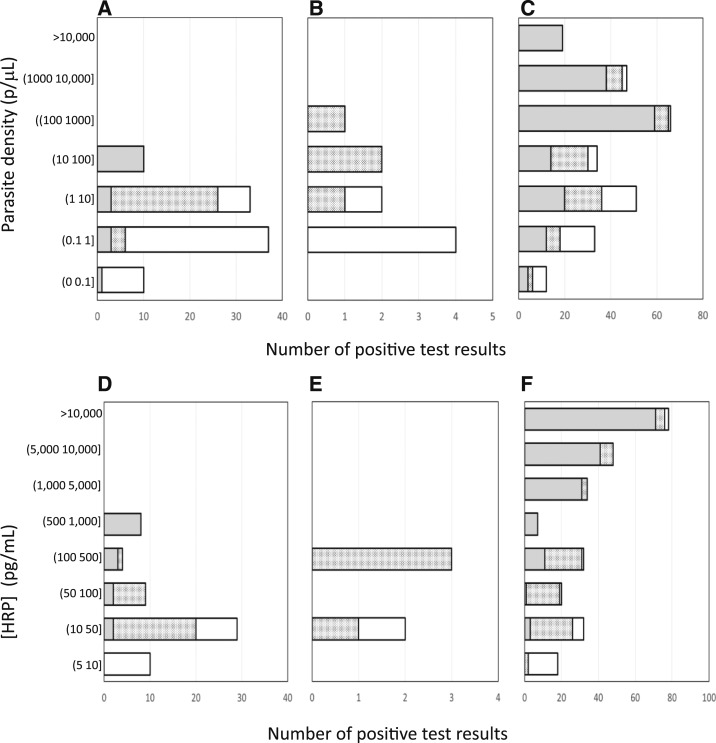
Distribution of specimens by parasite density and histidine-rich protein II (HRP2) concentration in induced blood-stage malaria challenge studies (**A**, **D**); Myanmar study (**B**, **E**); and Uganda study (**C**, **F**). (**A**–**C**). The outer clear bars represent the specimens that were positive by quantitative reverse transcription polymerase chain reaction; overlaid are the number of positive test results by ultra sensitive rapid diagnostic test (uRDT) (checkered bars) and by RDT (gray bars). (**D**–**F)**. The outer clear bars represent the specimens that were positive by quantitative HRP2 assay; overlaid are the number of positive test results by uRDT (checkered bars) and by RDT (gray bars).

From Days 4 to 7, 93 post-challenge specimens from 16 participants were tested by both qRT-PCR and Q-plex ELISA for *P. falciparum*. Of these, 92/95 (97%) were detected by qRT-PCR and 62/95 (65%) positive by Q-plex ELISA. Of the 92 qRT-PCR-positive specimens, 61 (66%) were also Q-plex ELISA positive. The parasite densities and HRP2 concentrations are shown in Figure 1A. The correlation between HRP2 concentration and parasite density is shown in Figure 1B. At low densities (0–5 p/µL), the correlation with HRP2 was weak (*R*^2^ = 0.45), but when extended to 2,200 p/µL, the correlation improved (*R*^2^ = 0.88 [data not shown]). The distributions of the specimens sorted by either parasite density or HRP2 concentration are shown in Figure 2.

#### TOT village, Myanmar.

A total of 124 specimens were positive for *Plasmodium* 18S rRNA consistent with infection by qRT-PCR, of which five (3.9%) specimens were *P. falciparum* positive only, four (3.1%) were *P. falciparum* mixed positive, 97 (78.2%) were *P. vivax* positive, and 18 (14.1%) were *Plasmodium* spp. positive. *Plasmodium vivax* positivity was determined from the qRT-PCR pan-*Plasmodium*-positive samples and the high-volume qPCR followed by nested PCR to specifically identify *P. vivax*. There were a total of nine (1.8%) *P. falciparum*-positive specimens (includes *P. falciparum* mixed infections) in the overall collection (*N* = 493). The parasite densities of the nine positive *P. falciparum* specimens by qRT-PCR ranged from 0.2 to 136.9 p/µL (Table 1) with a geometric mean of 2.9 p/µL (95% confidence interval [CI]: 0.4–20.4) and median of 1.2 p/µL. For the *P. falciparum* mixed infection specimens only, the qRT-PCR pan-*Plasmodium* spp. parasite density ranged from 58.2 to 927.0 p/µL with a geometric mean of 168.3 p/µL (95% CI: 4.1–6,856.2) and a median of 88.4 p/µL. The 96 specimens that were non-*P. falciparum* by qRT-PCR, and *P. vivax* positive only by qPCR, had a pan-*Plasmodium* spp. parasite density range of 0.11–15,697.2 p/µL with a geometric mean of 31.2 p/µL (95% CI: 20.1–48.6) and a median of 23.5 p/µL. The corresponding qPCR parasite densities for the *P. vivax*-infected specimens ranged from 0.5 to 32,724.4 p/µL with a geometric mean of 42.2 p/µL (95% CI: 27.5–64.7) and median of 37.4 p/µL.

**Table 1:** Parasite density and HRP2 concentration as well as RDT and uRDT results for the Myanmar data

Parasite desnity (p/µL)	HRP2 (pg/mL)	uRDT result	RDT result
136.9	183.2	+	−
76.4	265.6	+	−
14.1	31.2	+	−
9.2	0	−	−
1.2	151.0	+	−
0.8	0	−	−
0.3	0	−	−
0.3	0	−	−
0.2	0	−	−
0	18.8	−	−
0	0	+	−

HRP2 = histidine-rich protein II; uRDT = ultra sensitive rapid diagnostic test. Data are shown only for those results that were positive by at least one test modality.

The quantitative HRP2 assay was able to quantify HRP2 in five specimens from Myanmar, of which four were confirmed by *P. falciparum* qRT-PCR (Table 1). The HRP2 concentrations in these specimens were low, ranging from 31.2 to 265.6 pg/mL. The mean HRP2 was 157.7 pg/mL and median was 167.1 pg/mL. The highest concentration, 265.6 pg/mL, is much lower than the RDT LOD (800 pg/mL), but higher than the uRDT LOD (80 pg/mL). The distributions of the specimens stratified by parasite density or HRP2 concentration are shown in Figure 2.

#### Nagongera, Uganda.

*Plasmodium* species were detected and measured by qRT-PCR in 292 specimens, of which 249 (85.3%) were *P. falciparum* only positive, 12 (4.1%) were *P. falciparum* mixed, and 31 (10.6%) were non-*P. falciparum*. There were a total of 261 (43%) *P. falciparum*-positive specimens, including the *P. falciparum* mixed specimens, in the overall collection (*N* = 607). Quantification of *P. falciparum* parasite density revealed a wide distribution, 0.01–235,095 p/µL (Figure 2). The geometric mean parasite density was 54.3 p/µL (95% CI: 34.2–86.3) and the median was 100.2 p/µL. For the *P. falciparum* mixed infection specimens only, the qRT-PCR pan-*Plasmodium* spp. parasite density ranged from 1.2 to 16,126.9 p/µL with a geometric mean of 303.1 p/µL (95% CI: 52.5, 1,749.2) and a median of 367.2 p/µL. The 96 specimens that were non-*P. falciparum* by qRT-PCR had pan-*Plasmodium* spp. parasite density range of 0.09–1,373.5 p/µL with a geometric mean of 17.1 p/µL (95% CI: 7.0–42.0) and a median of 11.3 p/µL.

*Plasmodium falciparum* HRP2 was quantified in 269 specimens with concentrations ranging from 6.1 to 14,600 pg/mL (upper bound of the Q-plex dynamic range: 14,600 pg/mL) (Figure 2). Of the 269 specimens, 226 (84%) were also parasitemic by qRT-PCR. The mean and median levels of HRP2 were 5,614.2 and 4,122.9 pg/mL, respectively.

Thirty-five specimens were qRT-PCR positive but Q-plex ELISA negative; all of these had low parasite densities. The overall geometric mean for these specimens was 0.74 p/µL (95% CI: 0.37–1.5) and median was 1 p/µL. The distributions of the specimens sorted by either parasite density or HRP2 concentration are shown in Figure 2.

**Figure 3.**
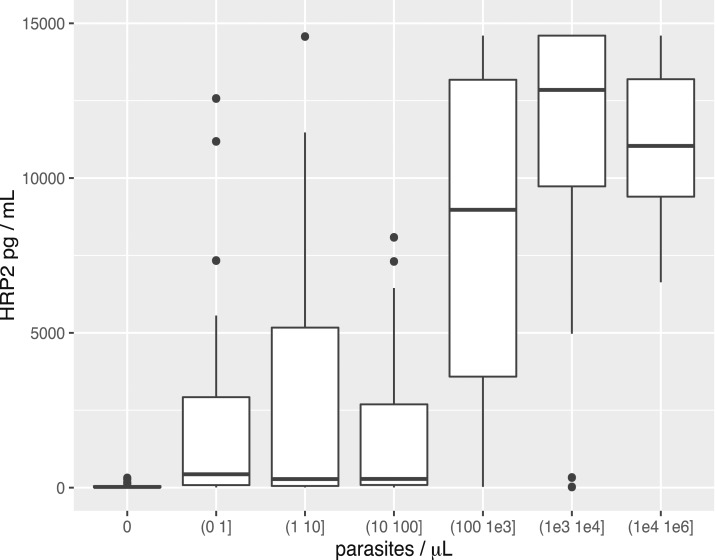
Relationship between parasite density and histidine-rich protein II (HRP2) concentration shown as box plots for ranges of parasite densities. The data shown are for the Uganda specimens. The upper and lower bounds of the boxes represent the third and first quartiles, respectively. The mean is indicated by a solid line and the whiskers indicate the 95th and 5th percentiles. The leftmost bin for 0 parasites per µL (p/µL) corresponds to HRP2-positive but quantitative reverse transcription polymerase chain reaction-negative specimens. The descriptive statistics are provided in Table 3.

The correlation between HRP2 and parasite density was poor, although the median and mean values tended to increase with parasite density (Figure 3 and Table 2).

**Table 2:** Descriptive statistics for the relationship between parasite density and histidine-rich protein II (HRP2) concentration, shown also as box plots in Figure 3

Parasite density (p/µL)	Median HRP2 (pg/mL)	Mean HRP2 (pg/mL)	Minimum HRP2 (pg/mL)	Maximum HRP2 (pg/mL)	First quartile HRP2 (pg/mL)	Third quartile HRP2 (pg/mL)
*0*	*22*	*49*	*5*	*314*	*10*	*50*
(0, 1]	430	2,216	6	12,570	83	2,918
(1, 10]	275	2,710	6	14,570	51	5,171
(10, 100]	278	1,721	8	8,085	85	2,692
(100, 1,000]	8,973	8,377	25	14,600	3,586	13,180
(1,000–1 × 10^4^]	12,850	11,630	21	14,600	9,730	14,600
(1 × 10^4^–1 × 10^6^]	11,040	11,080	6,635	14,600	9,398	14,600

The italicized row corresponding to 0 parasites per µL (p/µL) corresponds to HRP2 positive but qRT-PCR negative specimens.

**Figure 4.**
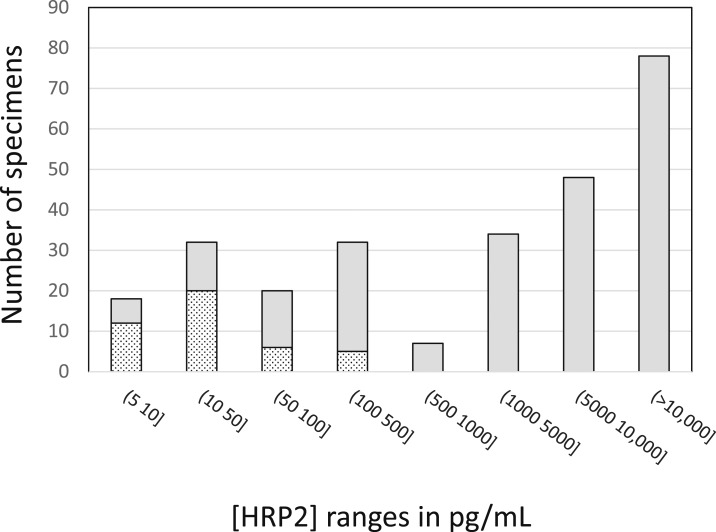
Histogram of Uganda asymptomatic clinical specimens arranged by histidine-rich protein II (HRP2) concentration ranges and broken out into confirmed parasitemic (gray shaded bars) and non-parasitemic (spotted bars) specimens based on quantitative reverse transcription polymerase chain reaction data.

There were also 43 specimens that were positive for HRP2 by Q-plex ELISA but *P. falciparum* negative by qRT-PCR (Figure 4). These specimens had low HRP2 concentrations, ranging from 5.1 to 313.7 pg/mL with median and mean values of 48 and 22 pg/mL, respectively.

### Performance of the RDT and uRDT.

The performance of the RDT and new uRDT was evaluated against specimens from the non-endemic site IBSM studies, Myanmar, and Uganda. All specimens had been previously frozen and stored at −80°C prior to application to the two different RDTs.

#### Pretreatment specimens in *P. falciparum* IBSM challenge studies.

Performance of the uRDT and RDT during early infection was evaluated in IBSM studies at the non-endemic Australian site, where preceding infections in study participants could be excluded. The uRDT was able to detect infections as early as 1.5 days sooner than the RDT during early onset infections (Table 3; *P* < 0.05 at day 5.5). The performance of the uRDT and RDT against confirmed parasite density and HRP2 concentration is shown in Figure 2 and summarized in Table 4.

**Table 3:** Positivity by the Q-plex HRP2 assay, uRDT, and the RDT for IBSM specimens with parasite density detectable by qRT-PCR

Day post-IBSM challenge	4	5	5.5	6	6.5	7.0
qRT-PCR	13	16	15	16	14	16
Q-plex HRP2 (%)	2 (15.4)	6 (37.5)	12 (80.0)	13 (81.3)	10 (71.4)	16 (100)
RDT (%)	1 (7.7)	1 (6.3)	2 (13)	1 (6.3)	0 (0)	12 (75.0)
uRDT (%)	1 (7.7)	1 (6.3)	10 (66.7)	7 (43.8)	7 (50.0)	16 (100)
*P* value RDT/qRT-PCR vs. uRDT/qRT-PCR	1	1	0.003	0.014	0.002	0.032

HRP2 = histidine-rich protein II; qRT-PCR = quantitative reverse transcription polymerase chain reaction; uRDT = ultra sensitive rapid diagnostic test. *P* values for the significance in difference between positivity in the RDT vs. that of the uRDT are provided.

**Table 4:** Performance of the RDT and uRDT

	Reference assay: qRT-PCR					Reference assay: Q-plex			
	Sens.	Spec.	PPV	NPV		Sens.	Spec.	PPV	NPV
Early infection IBSM studies									
RDT	19 (12–29)	100 (31–100)	100 (77–100)	4 (1–12)		25 (15–38)	94 (78–99)	88 (62–98)	40 (30–53)
uRDT	47 (36–57)	100 (31–100)	100 (90–100)	6 (2–17)		68 (55–79)	97 (82–100)	98 (86–100)	63 (48–76)
Myanmar (low transmission)									
RDT	0 (0–37)	100 (99–100)	NA	98 (96–99)		0 (0–54)	100 (99–100)	NA	99 (97–100)
uRDT	44 (15–77)	99.8 (99–100)	80 (30–99)	99 (97–100)		80 (30–99)	99.8 (99–100)	80 (30–99)	99.7 (99–100)
Uganda (high transmission)									
RDT	62 (56–68)	95 (92–97)	91 (86–95)	77 (73–80)		61 (55–67)	96 (93–98)	92 (87–95)	76 (71–80)
uRDT	84 (79–88)	92 (88–94)	88 (83–92)	88 (84–91)		91 (87–94)	99 (97–100)	98 (96–99)	93 (90–96)

NPV = negative predictive value; PPV = positive predictive value; RDT = rapid diagnostic test; qRT-PCR = quantitative reverse transcription polymerase chain reaction; Sens. = sensitivity; Spec. = specificity; uRDT = ultra sensitive rapid diagnostic test. The Sens., Spec., PPV, and NPV are listed as percentages for each set of study, using either Plasmodium 18S rRNA qRT-PCR results as the reference values or the Q-plex histidine-rich protein II enzyme-linked immunosorbent assay as the reference values. 95% confidence intervals are provided in brackets for each performance value.

#### TOT village, Myanmar.

The detection of *P. falciparum* HRP2 by RDT and uRDT was performed using the total collection of 493 whole blood specimens. Overall, 0/493 RDT-positive and 5/493 (1%) uRDT-positive specimens were detected. Almost half (4/9, or 44.4%) of qRT-PCR and all (4/4, or 100%) of Q-plex ELISA-confirmed *P. falciparum*-positive specimens were also uRDT positive, and one uRDT-positive specimen was neither qRT-PCR positive nor Q-plex ELISA positive. The details of qRT-PCR specimens in terms of parasite density, HRP2 concentration, and RDT and uRDT performance are shown in Figure 2 and Table 1. Only the uRDT was able to detect confirmed *P. falciparum* specimens, and both tests were equivalent in terms of specificity (Table 4).

#### Nagongera, Uganda.

All blood specimens from asymptomatic study participants (*N* = 607) were tested with both the RDT and uRDT. The prevalence of malaria infection by uRDT as compared with RDT was 40.7% (247/607) and 29.5% (179/607), respectively. Most of the positive specimens by uRDT (218/247, or 88.3%) and by RDT (163/179, or 91.1%) were also qRT-PCR positive, such that there were 29 uRDT- and 16 RDT-positive results confirmed as non-parasitemic by qRT-PCR, of which 26 uRDT and only four RDT were confirmed HRP2 positive by the Q-plex-ELISA. The details of qRT-PCR specimens in terms of parasite density, HRP2 concentration, and RDT and uRDT performance are shown in Figure 2. At the lowest range of parasite density, > 0 to 0.1 p/µL, the uRDT detected 50% (6/12) of qRT-PCR-confirmed infections (Figure 2). In contrast, 50% or greater of qRT-PCR-positive specimens were only detectable by RDT in the 100–200 p/µL range.

Using qRT-PCR as the reference standard, the sensitivity and specificity of uRDT are 84% (95% CI: 79–88) and 92% (95% CI: 88–94), respectively, in comparison to 62% (95% CI: 56–68) and 95% (95% CI: 92–97), respectively, for the RDT (Table 4). Using the Q-plex HRP2 assay as the reference test, the sensitivity and specificity of the uRDT are 91% (95% CI: 87–94) and 99% (95% CI: 97–100), respectively, in comparison to 61% (95% CI: 55–67) and 96% (95% CI: 93–98), respectively, for the RDT (Table 4).

**Figure 5.**
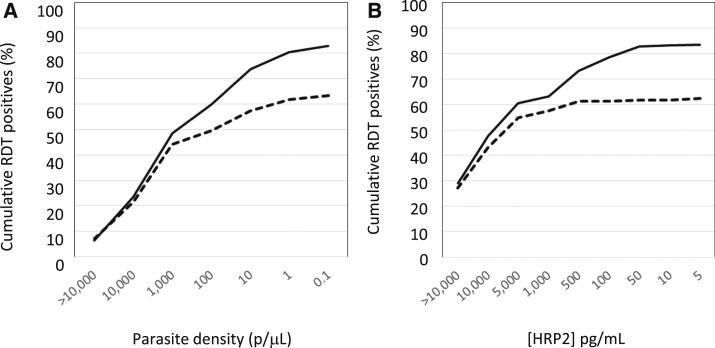
Cumulative proportion of the confirmed parasitemic asymptomatic specimens in the Uganda study that are uRDT (solid line) or RDT (dashed line) positive with (**A**) decreasing parasite density as determined by quantitative reverse transcription polymerase chain reaction (left to right) and (**B**) decreasing histidine-rich protein II (HRP2) concentration as determined by the HRP2 Q-plex enzyme-linked immunosorbent assay (left to right).

Figure 5 shows the cumulative proportion of confirmed qRT-PCR specimens that are RDT or uRDT confirmed, with decreasing parasite density or decreasing HRP2 concentration for the Nagongera data set. The RDT positivity rate plateaus at parasite densities below 100 p/µL in comparison to the uRDT that continues to rise with lower parasite density ranges (Figure 5 A). The uRDT detected over 50% of specimens with parasite densities between 0.1 and 1 p/μL, whereas the RDT only achieved this in the 100–200 p/μL range (data not shown). Similarly, below HRP2 concentrations of 500 pg/mL, the RDT has a lower detection rate than uRDT (Figure 5B). The uRDT detected over 50% of specimens with 25–50 pg/mL HRP2 concentrations, whereas the RDT only achieved this in the 400–500 pg/mL HRP2 concentration range (data not shown).

## DISCUSSION

These studies sought to determine the performance of a new highly sensitive RDT for *P. falciparum* malaria, the Alere Malaria Ag P.f. uRDT using specimens collected from different epidemiological settings, specifically for detecting asymptomatic infections. The performance was also evaluated in IBSM studies with malaria-naive study participants in the context of early infection HRP2 dynamics and time-to-infection detection in new infections. The performance of the uRDT was compared with that of currently commercially available SD BIOLINE Malaria Ag P.f. RDT (SD/Alere, Cat. 05EK40). The RDT has an LOD of approximately 800 pg/mL HRP2^25^ as compared with uRDT, which has an LOD of 80 pg/mL (Peck et al., 2017, manuscript in preparation).

The qRT-PCR data and Q-plex ELISA showed that in a high-transmission setting such as Nagongera, Uganda, there was a wider range of parasite densities and HRP2 concentrations in asymptomatic infections than in the low-transmission TOT village, Myanmar, where parasite biomarker concentrations were relatively low. The results for parasite densities are consistent with previous studies in low- and high-transmission settings.^6,20^ In Nagongera (high transmission), several immune mechanisms likely influence these varying levels of parasite densities.^13,34^ In low-transmission areas such as TOT village, where there have been rapid decreases in malaria prevalence, just two or three bites can form exposure-related immunity and suppress parasite loads.^21,34,35^ Many of the asymptomatic infections identified in these studies are likely due to previous exposure with parasite densities suppressed to submicroscopic levels.

The relationship between parasite density and HRP2 concentration is poor, as demonstrated previously.^36,37^ There are several possible factors that may explain these disparities, including parasite sequestration,HRP2 persistence, clonal variation in HRP2 expression, and prevalence of parasite strains with gene deletions in pfhrp2 or pfhrp3 or both. Sequestration occurs when *P. falciparum* trophozoites and schizonts adhere to vasculature and effectively disappear from peripheral blood circulation such that parasites cannot be visualized by microscopy.^38,39^ At the same time, HRP2 released during schizont rupture can remain in circulating blood during sequestration events, making it easier to detect by HRP2-based tests such as ELISAs and RDTs.^38^ Alternatively, HRP2 can also remain in detectable amounts after parasite clearance, resulting in “false-positives test results” from the perspective of the test result reflecting active infection.^10,24,40–43^

An HRP2-based diagnostic test produces false positives with respect to parasite density because either the specimen truly has residual HRP2 but no intact parasites or because the assay is erroneously detecting HRP2 where there is none; for example, cross-reaction with other proteins such as rheumatoid factor.^38,44^ The Q-plex HRP2 assay does not cross-react with rheumatoid factor (manuscript in preparation). In the Myanmar study, the Q-plex assay only detected HRP2 in one out of 484 qRT-PCR-negative specimens. In the Uganda study, however, there were a significant number of study participants who had detectable levels of HRP2 by the Q-plex assay but for whom active infection could not be confirmed by qRT-PCR. These HRP2-positive, qRT-PCR-negative samples all had low HRP2 concentrations and may support the presence of residual HRP2 in a high-prevalence setting.

The availability of a highly sensitive ELISA for HRP2, with different capture and detection reagents to the RDTs, as well as qRT-PCR facilitated the comparison of the performance of the uRDT and the RDT against both parasite density and HRP2 concentration in the blood. Notably, in Myanmar, the uRDT only detected one false positive in 484 confirmed negatives by qRT-PCR for a specificity of 99.7%. This specimen was also HRP2 negative by the Q-plex ELISA. In the Uganda study, 26 of the 29 non-parasitemic uRDT-positive tests were confirmed HRP2 positive by the HRP2 Q-plex ELISA, resulting in an improvement in specificity of the test from 92% as confirmed by 18S rRNA qRT-PCR to 99% by confirmed antigenemia (HRP2 Q-plex ELISA). In contrast, only four of 16 non-parasitemic RDT were confirmed HRP2 positive such that the specificity remained essentially the same, 95% and 96% compared with qRT-PCR and Q-plex ELISA reference assays, respectively. Thus, the uRDT is highly specific for individuals with asymptomatic infection or who have recently been infected.

By using the Q-plex ELISA, this study was able to confirm a more than 10-fold improvement in HRP2 LOD of the uRDT as compared with RDT using clinical specimens from asymptomatic patients. This improvement in LOD of the uRDT led to an increase in detection of asymptomatic cases in all the studies. In Uganda, with a *P. falciparum* prevalence of 43%, the sensitivity by uRDT was greater than that of RDT, 84% and 62%, respectively. In Myanmar, with a *P. falciparum* prevalence of 1.8%, the improved LOD of the uRDT resulted in the detection of 4/9 confirmed *P. falciparum* infections as compared with zero cases with the RDT. Both of the tests used in this study are based on HRP2 detection as such they will not detect parasites with pfhrp2 deletions. In the studies described here, there were no obvious high parasite density, negative HRP2 cases observed, that would be indicative of infections consisting of predominantly pfhrp2/pfhrp3 deletions. In locations where there is a confirmed high prevalence of pfhrp2/pfhrp3 deletions, this should be considered.^45–47^ Further studies are required in other settings with low-to-intermediate malaria transmission to better understand the performance of the uRDT with respect to the asymptomatic reservoir. The ability of the uRDT to detect infections earlier than the RDT, as demonstrated in the *P. falciparum* IBSM study, also adds further support to the utility of the improved uRDT LOD. The IBSM study showed that as early as 5 days after inoculation with infected erythrocytes, infection could be detected in naive individuals using the uRDT at significantly larger proportions than the RDT. In fact, on Day 6.5, 12 hours before treatment commenced, the RDT still detected no infections as compared with 7/14 infections by the uRDT. The impact of early detection as demonstrated in the IBSM studies on elimination programs in field settings will likely vary depending on transmission dynamics in a population at the time of screening.

The ability of the uRDT to detect large proportions of *P. falciparum* true positives relative to qRT-PCR and Q-plex ELISA results at all parasite LODs is encouraging for its potential use in malaria elimination settings. Low parasite density infections not detected by microscopy are potentially also infectious to mosquitos. Modeling data have suggested that when microscopy is used for malaria diagnosis in the field in a low-prevalence setting (10–20%), approximately 20–50% of asymptomatic individuals harboring parasites contribute to mosquito infection and continued transmission.^21^ With a diagnostic tool that has an LOD of 200 p/µL, it was shown in Burkina Faso that 55% of the infectious reservoir would be detected, followed by 83% at 20 p/µL and 95% at 2 p/µL.^21^ Although the studies in Myanmar and Uganda were unable to perform mosquito feeding studies to investigate the transmissibility of low-density infections, the uRDT demonstrated its ability to detect a greater number of individuals with low-density infections, and potentially also infectious individuals as compared with current field-based diagnostic tools such as microscopy and RDT. Future studies investigating infectiousness of the asymptomatic reservoir using mosquito challenge studies should be performed in combination with biomarker quantification as performed in the present study. Additionally, it would be valuable to study gametocyte density and dynamics in low-density infections and its contribution to oocyst and sporozoite development in the mosquito vector. Together, these results would support and improve model predictions of uRDT impact on different epidemiologies and elimination strategies.

Although the present study provided a comprehensive characterization of biomarkers and uRDT performance in asymptomatic infections from two regions with distinct endemicity and also in a challenge model for early infection dynamics, there were several limitations. The sample size for the Myanmar survey was too small to provide statistically significant sensitivity values in low-prevalence settings where most asymptomatic infections have very low parasite density. The blood specimens were collected after mass drug administration, which very likely contributed to low malaria prevalence in this area. Likewise, in the *P. falciparum* IBSM challenge study, specimens were collected from only 16 people at five time points, which resulted in a relatively small sample size for early infection analyses. Future studies should expand the survey to a larger sample size. Additionally, given that the IBSM study participants had no previous parasite exposure, it would be interesting to investigate and confirm early infection detection by uRDT in similar studies in malaria-endemic areas. In the Uganda study, the asymptomatic infection prevalence was too high to be considered as an elimination setting. Moreover, a majority of the Uganda specimens were from children, which may not be representative of the parasite density profile in asymptomatic infections in this setting. The Nagongera data, however, do provide useful information about biomarker levels and infection detection of asymptomatic subpopulations in high-transmission areas.

## CONCLUSION

The improved performance characteristics of the Alere Malaria Ag P.f. uRDT over the current malaria RDTs in asymptomatic patients from Myanmar and Uganda, as well as naive participants in the IBSM studies, are highly indicative of the utility of this test as a tool for malaria elimination. This performance should be verified under more operational conditions using finger stick blood specimens and performed by programmatic or health-care workers. Further studies and modeling are warranted to assess the impact and cost-benefit of the uRDT over other tests such as PCR in different malaria elimination strategies.
